# Patient perspectives and experiences with *in vitro* fertilization and genetic testing options

**DOI:** 10.1177/2633494119899942

**Published:** 2020-04-16

**Authors:** Erin Rothwell, Brandy Lamb, Erin Johnson, Shawn Gurtcheff, Naomi Riches, Melinda Fagan, Maya Sabatello, Erica Johnstone

**Affiliations:** Associate Vice President for Research, Integrity and Compliance, The University of Utah, 75 South 2000 East, Salt Lake City, UT 84112, USA; Department of Obstetrics and Gynecology, The University of Utah, Salt Lake City, UT, USA; Department of Obstetrics and Gynecology, The University of Utah, Salt Lake City, UT, USA; Utah Fertility Center, Pleasant Grove, UT, USA; Department of Obstetrics and Gynecology, The University of Utah, Salt Lake City, UT, USA; Department of Philosophy, The University of Utah, Salt Lake City, UT, USA; Department of Psychiatry, Columbia University, New York, NY, USA; Department of Obstetrics and Gynecology, The University of Utah, Salt Lake City, UT, USA

**Keywords:** *in vitro* fertilization, infertility, interviews, pre-implantation genetic testing

## Abstract

**Objective:**

Decision-making and patient experiences with embryo selection during *in vitro* fertilization often include genetic testing options. The purpose of this study was to gain insight about the experiences and perspectives of women using *in vitro* fertilization and genetic technologies.

**Methods:**

Interviews (*n* = 37) were conducted among female patients who had undergone *in vitro* fertilization, underwent expanded carrier screening, and were offered pre-implantation genetic testing for aneuploidy between July 2016 and July 2017. The interviews were transcribed and a content analysis was conducted on the transcripts.

**Results:**

Categories that emerged from the data analysis included unexpected outcomes, uncertainty, unanticipated emotional consequences, too much emphasis on the woman’s contributions and questions about embryo viability. Patient experiences with genetic technologies during *in vitro* fertilization played a significant role within these results.

**Conclusion:**

The emotional and psychological impacts of infertility during *in vitro* fertilization were the primary concerns discussed by participants. Future research is needed to identify ways to help manage unexpected outcomes and continuous uncertainty, including the increasing use of genetic technologies, to not add to the psychological burden of infertility. There is a need to explore more support options or counseling services for patients struggling with infertility during *in vitro* fertilization treatment.

## Introduction

Infertility is a growing public health problem in the United States.^1^ Identifying ways to help maintain and preserve fertility and promote prevention, early detection, and treatment of medical conditions that threaten fertility are a national priority.^2,3^ The CDC National Survey of Family Growth (2011–2015) determined that 6.7% of American women of reproductive age are infertile, and 7.3 million have used infertility services.^4^ This increase is partially due to larger numbers of couples seeking relational and economic stability before having children, thus prolonging the time before they attempt to start a family. However, emerging research is also suggesting that there are additional factors that are related to infertility such as genetics (e.g. endometriosis or an inherited chromosome abnormality such as Klinefelter syndrome), environment, infectious agents, and lifestyle factors that play significant roles.^2,3,5,6^

The psychological impacts related to *in vitro* fertilization (IVF), not specific to the associated genetic technologies, have been fairly well studied. Infertility treatment is physically, emotionally, and socially difficult,^7,8^ and there are psychological costs that are often overlooked as infertility is treated as a medical condition.^9^ Besides social stigma, studies indicate that many women seeking assisted reproductive technologies (ART) suffer from depression, anxiety, and distress.^10^ Some studies also found that infertile couples have lower marital satisfaction than fertile couples.^11,12^ Still other studies indicate that infertile couples are generally mentally healthy, however, coping with infertility is associated with heightened levels of negative psychological symptoms.^13^ Finally, although some insurance companies in the United States have started covering ART and some states mandate IVF coverage,^9^ costs of IVF are expensive, adding an additional burden to the process.

More research is needed to understand the psychological impacts of infertility, particularly as they relate to genetic testing and the decision-making process.^14,15^ Increased use of genetic testing alongside IVF and other infertility treatments raises new questions about the psychological impact of genetic technologies. Three highly used genetic testing practices in IVF today are expanded carrier screening (ECS), pre-implantation genetic testing for Mendelian disorders (PGT-M), and pre-implantation genetic testing for aneuploidy (PGT-A).^16^ For example, a common practice with IVF is for all couples to undergo ECS prior to beginning treatment.^17^ ECS can identify a couple at risk for a heritable disease that may help explain difficulties in achieving pregnancy and may lead the couple to add genetic embryo testing. One type of genetic embryo testing is PGT-M, which is available for fertile or infertile couples who want to avoid transmitting a detectable genetic condition. PGT-M has been relatively accurate for a single disease and most commonly used by couples whose offspring are at risk for a heritable disease. The number of diseases PGT-M can detect has rapidly increased.^18^ Another type of genetic embryo screening is pre-implantation genetic testing (PGT-A) which is chromosomal screening for aneuploidy. PGT-A screens for the presence of too few or too many chromosomes from a trophectoderm biopsy taken from a blastocyst on Day 5, 6, or 7 of embryo development. This technology is increasingly being used as a screening tool for all couples undergoing IVF to aid in embryo selection.^19^

Despite the increasing utilization of PGT-A, the evidence of its effectiveness for improving live birth rates is still inconclusive.^20,21^ Genetic embryo testing may be particularly appealing as it may increase couple involvement in the embryo selection process, allowing them to have some input on deciding which embryos to transfer. Some have argued that this increased involvement of the couple in embryo selection has changed models of autonomy in decision-making by moving to the front a shared relational approach between providers and couples, but more research with patient preferences with decision-making is needed.^22^ In light of these emerging technologies and changing paradigms of decision-making with embryo selection, exploring how genetic testing may or may not influence experiences is reasonable.^23^ The purpose of this study was to gain insight about the experiences and perspectives of women using IVF in association with one or more of these genetic technologies (those being ECS, PGT-M, and PGT-A).

## Methods

### Study sites and interview guide

A retrospective chart review was conducted to identify women who had undergone IVF in the previous year, underwent ECS, and were offered PGT-A between July 2016 and July 2017. This study only included women for two reasons. First, patient contact information retained by the clinic is for the woman undergoing IVF. Furthermore, many of the clinicians stated that the woman is primarily the one engaged in decision-making for which course of treatment to pursue during IVF. However, future research will need to include partner perspectives to assess how couples as opposed to women perspectives experience ART. Two sites were used for data collecting and included a for-profit and an academic clinic. PGT-A was chosen for focus for this particular study due to the increasing use of this testing compared with PGT-M. A semi-structured interview guide was created from a review of literature and expert input. The interview guide was designed to capture various aspects of patient experiences and perspectives of the IVF process and questions included: Why did you pursue IVF?; Throughout the IVF experience, did you have any surprises or unexpected outcomes?; Looking back on your journey with IVF, is there anything particular that has stood out to you?; What do the genetic test results mean to you? What would you have liked to have known before beginning IVF?; Looking back, what was most helpful in understanding the process of IVF? Institutional review board at the University of Utah approved this study and patients were consented into this study (IRB #98692). The results presented here are independent but complement the other goals of the parent study around decisional factors to accept or decline PGT-A with IVF.^24^ The results presented here focus on participants’ reports of their experience with IVF and ECS, with or without PGT-A.

### Recruitment and participants

Letters were mailed to potential female participants from two fertility clinics. All prospective participants were patients who were within 3 to 12 months of an embryo transfer and had been offered PGT-A but may or may not chose to use PGT-A. Both an academic clinic and a private clinic were used (*n* = 100, 50 from each clinic). The two clinics did not differ in terms of treatment availability, when PGT-A was offered, or which provider introduced PGT-A to the patient (the MD). A pre-paid postcard was included in the mailings for participants to return indicating they would or would not participate in the interviews. Approximately, 30% returned postcards (two of which indicated a choice to opt out). For those who did not return a postcard, one or two additional attempts were made with a telephone follow-up approximately 2 weeks after the initial letter was mailed. The total response rate, excluding the five respondents who were unable to be contacted for interviews after opting-in, was 37%. Participants who agreed to an interview gave verbal consent over the phone and to have the interview audio recorded. Thirty-seven interviews were conducted in total. All of the participants completed ECS and 21 had PGT-A on their embryos and remaining 16 were offered, but declined PGT-A. On average, interviews lasted 40 minutes. Each respondent who completed an interview was given a $40 gift card for her participation.

The average age of the participant was 37 years with an age range of 27–44 years. Most participants (97%) had health insurance, most were college educated (86%), and a majority (64%) reported household income over $75,000. Only four participants were currently living outside of Utah and four were not married. There were no significant differences between the two groups in demographics. The remaining demographic data, including race/ethnicity and outcomes of their most recent IVF cycle for all participants, are provided in Table 1.

**Table 1.** Demographic data (*n* = 36; 1 missing).

Age (mean)		36.86 years
Race/ethnicity	Caucasian	32 (88.89%)
	Chinese	1 (2.78%)
	Hispanic	1 (2.78%)
	Mixed/Other	2 (5.56%)
Marital status	Married	32 (88.89%)
	Divorced	2 (5.56%)
	Single	2 (5.56%)
State of residence	Utah	32 (88.89%)
	Idaho	2 (5.56%)
	Oregon	1 (2.78%)
	New Jersey	1 (2.78%)
Highest level of education	High school diploma	2 (5.56%)
	Some college	1 (2.78%)
	Associate’s degree	2 (5.56%)
	Bachelor’s degree	16 (44.44%)
	Graduate degree	13 (36.1%)
	PhD	2 (5.56%)
Household income	$100,000 or higher	17 (47.22%)
	$75,000 to $100,000	6 (16.67%)
	$50,000 to $75,000	8 (22.22%)
	$25,000 to $50,000	3 (8.33%)
	Less than $25,000	1 (2.78%)
	Pass	1 (2.78%)
Health insurance	Yes	35 (97.22%)
	No	1 (2.78%)
Outcome of most recent IVF cycle	Live birth	14 (38.89%)
	Currently pregnant	7 (19.44%)
	Waiting for embryo transfer	2 (5.56%)
	Not pregnant or failed cycle	12 (33.33%)
	Not pregnant (waiting for surrogate)	1 (2.78%)

IVF, *in vitro* fertilization.

### Coding and data analysis

A qualitative descriptive design was used for this study.^25,26^ This type of research examines research questions about discovering the context of experiences and gaining insights for understudied phenomenon using descriptive methodologies.^25^ All of the telephone interviews were audio recorded, transcribed by a professional transcription company, and verified for accuracy by one of the researchers. A content analysis was used to analyze the transcript data. A distinguishing feature of qualitative description analysis is to let the data guide the coding and to use the participants’ own words when possible to describe the phenomenon.^26^ The first five transcripts were reviewed along with the interview questions to create a coding template (see Table 2). The coding template then was systematically applied to all of the transcripts by one of the researchers (BL) and were reviewed and verified independently by another researcher (ER). No major discrepancies were identified.^27,28^ The frequency of codes was used to guide the development of categories. The codes were linked together based on similarity and summarized to identify the most frequently reported codes across and within each of the interviews.^27^ A qualitative software program called Dedoose was used to manage the analyses.^29^

**Table 2.** Representative questions in the semi-structured interview guide.

	Interview question	Related probes
Communicative experience	Tell me about how you first heard about IVF	Who was involved in this initial communication about IVF?
		After you heard about IVF, where else did you look to learn more?
		How would you describe the role of the provider in this process?
	What factors influenced your choice to pursue IVF?	What else did you consider besides IVF?
		What about IVF had you hesitant to pursue this method?
	What type of educational materials did you receive before IVF?	Where was the information from?
		How would you describe how useful these materials were in making a decision?
		What kinds of information/material were most useful to you and why?
	Where are you in the process of IVF?	In your own words, can you describe the IVF process up until this point?
	Looking back, is there anything about the process that stood out to you?	
	Do you remember if you had any type of genetic testing on the embryo before the transfer?	
	What type of education did you receive about PGS during your IVF experience?	
	Can you tell me why you did/did not choose PGS?	What was the most important factor in this decision?
		What other factors influenced your decision to pursue PGS?
	Were there any surprises or unexpected outcomes during your IVF experience?	Was the surprise the result of possible outcomes not being fully explained?
		Was the surprise due to anticipating that this outcome would not happen to you?
Developmental questions and perceptions	If you were to describe the process of combining egg and sperm and then the development of an embryo, how would you do that?	What does it mean to say that an embryo ‘develops’?
		Please describe your understanding of human development from embryo to birth
		Did your understanding change after the experience of IVF or PGT-A?
	What is the role of genetics in the process of embryo development?	
	What is your understanding of the term ‘genetic risk’?	Did this understanding change after pursuing PGT-A?
		What was your understanding before screening?
	With how much certainty do you think that the number of chromosome predicts outcomes for the embryo?	For example, would having an extra or missing chromosome affect how the embryo develops?
	Is it possible for an embryo to have chromosomal abnormalities and not develop any disease symptoms or the associated syndrome?	For example, is it possible for an embryo to have three copies of chromosome 21 and not have the associated features of Down syndrome?
		Did your provider discuss uncertainty concerning outcomes?
	How important do you think the environment is in determining outcomes for the embryo?	What do you think are the most important environmental factors for embryonic development?
Improvements	What would you have liked to have known before beginning IVF?	What information would have made the decision to pursue IVF easier?
		What information would have been the most useful or beneficial?
	What was most helpful to you in understanding the process of IVF? What was the least?	What about your experience would you like to keep the same? What would you change?

IVF, *in vitro* fertilization.

## Results

The results presented below include similarities across all of the interviews and did not focus on differences. The most frequently reported codes across interviews were grouped into five categories. These categories included unexpected outcomes, uncertainty, unanticipated emotional consequences, too much emphasis on the woman’s contributions, and questions about viability. We summarize each, with additional details on women’s experiences with genetic technologies during IVF.

### Unexpected outcomes

One of the most frequent comments by participants was the prevalence of unexpected outcomes while undergoing IVF. Interviewees described unexpected outcomes as occurring at various stages of the process, including but not limited to unanticipated results. Frequently mentioned in this regard were the number of embryos available for transfer, delays in transfer of an embryo, and outcomes of pregnancy. For example, one participant reported she was shocked by the limited number of available embryos and stated, ‘*After two and a half years, we only ended up with two viable embryos. The first one was a girl, we lost. The second one is the boy that we now have who is three and a half months old*’. Some participants expressed unexpected outcomes regarding the role of the genetic testing for aneuploidy in particular. Many participants discussed their surprise about the limited number of embryos available after PGT-A for transfer and their outcomes. For example, one participant stated, ‘*I thought I was gonna get four more healthy embryos to transfer and all of them came back with abnormal chromosomes* [PGT]’.

Other representative quotes that encapsulated unexpected outcomes included, ‘*There ended being a lot more to it that they had told me at the consultation. Once we actually got started, I didn’t realize like oh I have to be on birth control for like two months*’ and ‘*We just did not really know what we were getting ourselves into*’. One participant stated she did not even know this was possible but she had an unexpected delay in the transfer: ‘*My lining wasn’t thick enough-you have to wait*’. However, not all unexpected outcomes were unwelcomed. A few participants reported experiences such as ‘*Ours* [IVF process] *went incredibly easy. We conceived the first try, so that was nice*’.

### Uncertainty throughout the process

The second category of results that emerged from the data analysis is that the IVF process entailed continuous uncertainty that holds a significant emotional and financial burden. Even for participants who had undergone at least one cycle of IVF, they stated that each cycle is different and there are no guarantees within each cycle. Some representative quotes are as follows: ‘*Oh my gosh. My entire life’s happiness depends upon this next appointment*’; ‘*Going through everything, and all of the medications and everything, and then just not knowing if it was going to work or not, was the hardest thing*’; and ‘*We went through three rounds of egg retrieval and several canceled transfers after that due to my body just not responding to the hormones. It’s just the uncertainty*’.

There was also uncertainty with costs and how to manage the costs. For example, ‘*The costs. I knew it was expensive, but I didn’t realize that you have to pay upfront however much it costs*’; ‘*It’s mostly the financial piece. It’s so confusing and it’s so hard to know what fees are covered and what you pay already and what’s gonna be extra*’; and ‘*Our doctor was very honest that you could go through that whole process and pay all that money and still end up with nothing. That was scary*’.

### Psychological and emotional consequences

Beside the emotional burden of uncertainty, participants in our study expressed emotional and psychological consequences emerging from the challenges of infertility. Some representative quotes include the following: ‘*Most difficult thing ever*’ and ‘*I just think that like the emotional component of it is – it was more intense than even I had anticipated it to be*’. Others did not anticipate (and were not prepared for) their own responses to the IVF process, including that there was also a long-term emotional impact. Some participants expressed, ‘*Then I wasn’t prepared for it* [depression] *to stick around after I got pregnant. I thought I would be just fine. I guess it took my body a long time to clear it. I was super, super depressed for the first part of my pregnancy*’; ‘*I developed anxiety. I’m guessing a lot of women do. I don’t think of myself as an anxious person. I basically turned into somebody else, I had to take all these hormones that basically made me feel like a different person*’; and *‘For me, I felt like I was in a different body with a different brain for quite a while*’. Even individuals who achieved pregnancy stated similar comments but stated that commitment was worth it for them. For example, ‘*It was probably one of the hardest things I’ve ever done, just physically, emotionally, mentally, everything. It was a little rough, but it was definitely worth it in the long run*’.

In addition, many participants were not anticipating the strong emotional reactions to failed transfers. This was also discussed in terms of the lack of emotional support or counseling for failed attempts, miscarriages, or no successful transfers. Representative quotes included the following: ‘*So like with our miscarriage and failed cycles, the nurse kind of just say, ‘Yep sorry didn’t work out. When do you want to start your next cycle?’ the mental and emotional aspect of all of that is kind of ignored*’; ‘*I think for IVF, I think it’s – I feel like it’s a lonely process*’; and ‘*He was six rounds* [son]. *We didn’t find the support group until round three is when we found the support group, and that made the other three rounds much easier to handle*’.

### Too much pressure on the woman

An interesting category emerged around the pressure many of the participants stated they felt from the IVF process. Participants placed pressure on themselves to get pregnant, but many stated that they felt pressure by the clinic to get pregnant. When they did not get pregnant, the clinic suggested that the reason was primarily because of the woman’s lack of fertility and it promoted feelings of failure in some participants. Furthermore, many participants stated that the male’s contribution should be addressed more thoroughly early on and that this was not just a women’s problem. Quotes that captured this perspective included the following: ‘[Clinic staff] *They talk about who you are by what you produce* [quality of eggs]’; ‘*There was little discussion on how this may be the male*’; and ‘*I feel like a failure*’. Other quotes include the following:

*Because it’s really hard to not have hope, and it’s hard to go through that and feel like your life is reduced to these results that aren’t favorable. You’re always getting measured by how many follicles they have, and when there aren’t any, it’s hard to deal with it*.

*I think the more you get measured, and you’re not meeting your expectations or the results that they hoped for, it puts more stress on you. You over think it, and you start feeling like a failure. My body became more stressed, and it made it worse for me*.

### Confusion about results

Another frequent response concerned interpretation of returned results. Participants who received results from PGT-A were told that the embryos were either normal or abnormal but many did not know what that meant. Also, participants made comments about why there were not options in the middle. ‘*Nothing in between viable or not. Why?*’ Another quote that captured this category included the following:

*One of our embryos came back as abnormal. They weren’t sure. When we talked to the doctor, they weren’t sure what the abnormalities were. I guess that was a little bit confusing. They said they don’t know enough about it yet. All we know is we have a frozen embryo that’s abnormal. We don’t know what the abnormality is*.

PGT-A was not the only source of confusion about results. Across all participants, the results on how the embryos were growing were also confusing. In particular, participants were confused about information on the quality of the embryo. Two quotes that captured these concerns are given below:

*We were waiting to hear back. Are they growing? How many are we going to end up with? This guy called and said, ‘I was just calling to say of the 14, all of them died but 3. Any questions?’ I was like, ‘what?’ He said, ‘They just stopped growing’. I was like, how does that happen? He couldn’t explain it. I don’t know what that means. I just know that two hours ago we were thinking fourteen and now we’re thinking three.*

*I have no idea what you’re talkin’ about with the embryos. Like AA, B’s, whatever it is, and I said, I have no idea what you’re talkin’ about*.

## Discussion

There have been a number of studies to assess the psychological impacts of infertility, IVF, and ART.^30–34^ However, research that captures in-depth qualitative experiences and perspectives about IVF in conjunction with genetic technologies is scarce. This study addresses the gap by providing more descriptive data about experiences and perspectives with IVF and genetic testing. Our results corroborate earlier findings that the emotional and psychological consequences of infertility are high and can emerge from throughout the various components of the IVF process.^9,35^ In addition, the detailed responses of interview participants indicate that unexpected outcomes and continuous uncertainty are important psychological stressors, with implications for mental health and patient well-being. A recent study found clinically significant levels of depression or anxiety at some point during their IVF treatment.^34^ This is supported by earlier literature that mental health is a significant concern for fertility treatment.^36–38^ A study on experiences with pre-implementation genetic diagnosis (PGD) in Sweden found that even after three years after undergoing PGD, couples are still psychologically affected by the experience.^39^

These burdens can also result in negative mental health outcomes and can be the major reason for discontinuing IVF treatment.^15,40^ For example, research demonstrates that the emotional stress of fertility treatment is one of the main reasons patients with increased chances for pregnancy discontinue treatment prematurely.^41^ However, it is important to note that infertility in itself is often perceived as a life crisis where the emotional strain equals that found in traumatic events.^42,43^ The addition of IVF adds to these stressors but it is not the cause. A few participants in our study discontinued the IVF process due to both the additional negative stressors and financial costs. Our research adds to the literature that along with the burden of living with constant uncertainty, unexpected outcomes – both in terms of the limited embryos available for transfer and associated costs – can add to the stress that comes along with medical interventions.^30,33^ Genetic technologies add to this psychological burden, increasing the scope of uncertainty and opportunities for unexpected outcomes and costs. As noted in other research, the impacts of PGD on IVF treatment found that PGD does increase anxiety through both unmet information needs, costs, and emotional burden, which deter some couples from IVF.^44,45^

Patients undergoing IVF are being asked to make decisions about their embryos that they may not feel prepared to make based on limited understanding of the genetics or the test being offered. For instance, interviewees in this study were told there were abnormalities but they did not understand what that meant and what they were supposed to do with that information, especially when all the embryos turned out to be abnormal based on the PGT-A screen. A better understanding of the complexity of these stressors associated with genetic testing is needed to improve the clinical care of those struggling with these experiences and in light of the significance of mental health concerns during treatment.

The pressure that many of our participants expressed regarding the woman’s responsibility (or failure) to achieve a pregnancy appears to have further augmented the frustration with infertility. Participants stated that their lack of pregnancy made them feel like failures because they could not produce enough follicles or they miscarried. These emotional burdens were not anticipated by many participants. Although IVF clinics typically offer programs to assist mental health, many participants seem unaware of these programs, or chose not to take advantage of them. In this regard, our findings suggest that a parallel and complementary process aimed at reducing psychological stress of IVF may be needed throughout the entire IVF process, not as an option presented only at the beginning. Interesting, these findings support other research in that there are low rates of both referrals to mental health services by IVF clinics and lack of patient receiving mental health services despite the association of reduction of stress from infertility and mental health services.^33,36,37,39^ We conclude with two proposals for such a process.

While the emotional consequences of fertility treatment appear to be the primary concern at this time, there are lingering issues related to the growing involvement of genetic testing in the IVF process as well as embryo selection that may be based on uncertain genetic findings. Participants in our study expressed confusion about the process of embryo selection, the criteria by which viability is determined as well as questions about what makes an embryo abnormal and how that limits the embryos available for transfer. Ensuring that patients undergoing such an already challenging process are well informed about the results and provided with sufficient information to grapple with the outcomes is necessary. More guidance on how to communicate this new information into an already complex IVF process should be further explored.

Finally, there is a need for identifying ways to better communicate options for mental health services throughout the entire IVF process, especially after failed transfers or miscarriages. This may include rethinking how ‘bad news’ is communicated within the patient–provider relationship, reconsideration of the role of men and women in reproduction, and how biases about female’s role may exacerbate anxiety, and training to ensure that communication methods emphasize empathy and humility.

The limitations of this study design are the limited number of participants, the diversity of participants (drawn from only two clinics), and the inclusion of women only for interviews. Future research will need to examine specifically how attitudes toward use of genetic technologies during IVF differ by gender.^46^ Another is the potential for self-selection among participants, such that those who chose to participate wanted to discuss their concerns bearing on psychological effects of IVF with genetic technology.

## Conclusion

This study conducted in-depth interviews with 37 women who underwent IVF with ECS. All were offered genetic PGT-A with their treatment; 57% chose this option. Our interview guide aimed to discover how increased genetic testing options may have changed or influenced women’s experience of the IVF process. The emotional and psychological impacts of IVF on top of struggling with infertility were the primary concerns discussed by participants. There is a need to explore more support options or counseling services for women and couples struggling with infertility during IVF treatment. These will be even more needed as genetic testing becomes more prevalent in infertility treatment processes.

### Practical implications

As our study indicates, participants often experience IVF, including the genetic aspects, as a difficult process. Furthermore, there appeared to be little support or counseling services provided for patients – or these participants did not use the services recommended or provided by the clinic. These participants may not recognize the need for counseling services when they initiate the process. If participants seek support on their own, it may add to the financial burden, making additional costs for psychological support not feasible. There is a clear need for further research to better understand how to reduce the emotional and psychological burden of infertility and treatments for infertility, with low-cost options (e.g. support groups). Further research is also needed to ensure that services are effective, properly communicated, and do not add to the psychological burden of infertility.
